# Impacts of Chinese national centralized volume-based procurement policy targeting meropenem on prescription of designated antimicrobials for inpatients: an interrupted time series analysis

**DOI:** 10.3389/fphar.2025.1458792

**Published:** 2025-02-21

**Authors:** Zhi-Hang Lin, Can-Ming Wang, Li-Li Cai

**Affiliations:** Department of Pharmacy, Quanzhou First Hospital Affiliated to Fujian Medical University, Quanzhou, China

**Keywords:** ARIMA, interrupted time series analysis, meropenem, segmented regression, volume-based procurement

## Abstract

**Objectives:**

A national centralized volume-based procurement policy (NCVBPP) targeting meropenem has been implemented in China since December 2022. Here, the effects of the meropenem NCVBPP upon the prescription of designated antimicrobials for inpatients were explored.

**Methods:**

The impacts of the meropenem NCVBPP on the consumption of and expenditures for designated antimicrobials prescribed for inpatients were evaluated by means of an interrupted time series analysis (ITSA) using both an autoregressive integrated moving average (ARIMA) model and a segmented regression model. The designated antimicrobials consisted of carbapenem-type antimicrobials and carbapenem-replaced antimicrobials; the latter referred specifically to combinations of penicillins/cephalosporins with beta-lactamase inhibitors and cephamycins. Data on the consumption of and expenditures for designated antimicrobials used in the inpatient sector of our hospital during the period ranging from January 2020 to March 2024 were collected and subjected to an ITSA.

**Results:**

The meropenem NCVBPP boosted the consumption of meropenem (generic drug and original counterpart); however, neither the total consumption of carbapenem-type antimicrobials nor that of carbapenem-replaced antimicrobials was affected by the meropenem NCVBPP. On the other hand, the meropenem NCVBPP significantly decreased the expenditures on meropenem. Its impacts on the total expenditures for carbapenem-type antimicrobials were unknown. Although a transient increase in the expenditures for carbapenem-replaced antimicrobials and a reduction in the overall expenditures for carbapenem-type antimicrobials plus carbapenem-replaced antimicrobials were also observed following the meropenem NCVBPP, these results were not necessarily caused by the meropenem NCVBPP.

**Conclusion:**

The meropenem NCVBPP triggers increased consumption of but reduced expenditures for meropenem. It has no effects on either the overall consumption of carbapenems or carbapenem-replaced antimicrobials.

## 1 Introduction

In China, a national centralized volume-based procurement policy (NCVBPP) targeting drugs has been issued and implemented since 2018, with the main aim of reducing the prices of numerous medications on the premise of ensuring that they are quality assured (State Council of the PRC, 2019; Zhu et al., 2023). Almost all of the drugs adopted as procurement subjects of drug NCVBPPs are generic drugs. In most cases, a generic drug could be viewed as a candidate procurement subject of a drug NCVBPP only after it has been certified by a generic drugs consistency evaluation (State Council of the PRC, 2019). It has been shown that the intended price cuts of drugs covered by drug NCVBPPs were achieved following the drug NCVBPP (Yuan et al., 2021). Moreover, with the implementation of drug NCVBPPs, the utilization of the generic drugs subject to drug NCVBPPs and the rates of their substitution for the corresponding original drugs both markedly increased (Wang et al., 2022; Yang et al., 2023).

An NCVBPP targeting meropenem was launched in December 2022. Meropenem belongs to carbapenem-type antimicrobials, which, as broad-spectrum beta-lactam antimicrobials, are typically reserved as antimicrobials of last resort (Papp-Wallace et al., 2011; Armstrong et al., 2021). Meropenem is the most used carbapenem in healthcare institutes in China in 2022, the consumption of which (expressed as the number of defined daily doses (DDDs)) accounted for up to 52% of the total consumption of carbapenems (National Health Commission of the PRC, 2023).

Meropenem is well-tolerated and active against a wide range of Gram-positive and Gram-negative bacteria, including many resistant strains (Baldwin et al., 2008). It also has good tissue penetration (Craig, 1997). These characteristics of meropenem make it a preferred powerful weapon in the fight against numerous severe infections, such as those caused by multidrug-resistant organisms. In terms of pharmacoeconomics, meropenem, when given by intravenous bolus injection, has been shown to have lower total drug administration costs than imipenem/cilastatin. Compared with combination therapy involving the use of non-carbapenem antimicrobials, monotherapy with meropenem for serious infections may offer not only the potential for more efficient patient management but also the potential for cost savings (Holliday and Benfield, 1998).

Although the meropenem NCVBPP was expected to reduce the use cost of meropenem, its effects on the overall utilization and expenditures for carbapenem-type antimicrobials are currently unknown. There is a concern that the meropenem NCVBPP is likely to elevate the total consumption of carbapenem-type antimicrobials due to the improved accessibility and affordability of meropenem following the meropenem NCVBPP. In addition, for the same reason, the use of other types of antimicrobials with similar antimicrobial spectrums to carbapenems that generally take priority over carbapenems during the drug selection for the treatment of related infectious diseases (herein termed carbapenem-replaced antimicrobials) may also be affected by the meropenem NCVBPP. These carbapenem-replaced antimicrobials include combinations of penicillins/cephalosporins with beta-lactamase inhibitors and cephamycins. Whether the emergence of price-cutting meropenem induced by the meropenem NCVBPP would encourage clinicians to shift their prescription preference for the initial treatment of associated infections to carbapenems over carbapenem-replaced antimicrobials and thus lead to the decreased usage of carbapenem-replaced antimicrobials is a topic deserving study.

The present study was conducted to address the abovementioned concerns. Specifically, an interrupted time series analysis (ITSA) method was adopted in this study to reveal the implications of the meropenem NCVBPP for the consumption of and expenditures for both carbapenem-type antimicrobials and carbapenem-replaced antimicrobials used in the inpatient sector of our hospital.

## 2 Materials and methods

### 2.1 Data collection

Specified types of antimicrobials consumed by inpatients of our municipal comprehensive hospital were the antimicrobials of interest. These antimicrobials included carbapenems, combinations of penicillins/cephalosporins with beta-lactamase inhibitors, and cephamycins. The monthly data on both consumption (expressed as DDD/1000 people/day) and expenditures (expressed as thousand yuan) of antimicrobials of interest during the study period from January 2020 to March 2024 were collected. The implementation of the meropenem NCVBPP started in our hospital in December 2022. Therefore, there were 35 data points collected for the pre-NCVBPP period and 16 data points collected for the post-NCVBPP period. In other words, data collected during the period from January 2020 to November 2022 were used as a baseline, and data collected during the period from December 2022 to March 2024 were regarded as post-NCVBPP data. The baseline period was determined based on two major considerations. First, the baseline period should be long enough for the employed models to fully capture the data features. Second, January 2020, but not an earlier time, was chosen as the origin of the baseline period, as the outbreak of COVID-19 at the end of 2019 was thought to be a potential confounding factor in this study. All data were completely collected; no values were missing from any time series to be analyzed.

### 2.2 Statistical analysis

An ITSA using an ARIMA model and an ITSA using a segmented regression model were simultaneously adopted to analyze the data. The ARIMA model’s ability to handle autocorrelation, seasonality, and non-linear trends likely to be present in data of time series, combined with the segmented regression model’s strength in coping with autocorrelation, make these two models suitable to be employed in the ITSA (Box et al., 2015). R software (version 4.3.1) was employed to construct the ARIMA model according to previously published methods (Schaffer et al., 2021). Of note, the auto.arima() command in the forecast package for R was used to automatically identify the ARIMA model. The following settings were followed when implementing the auto.arima() command: a) Seasonal ARIMA model construction was requested, and the length of the seasonal cycle was 12. b) Two variables representing level change and slope change, respectively, were created and utilized in model construction. c) The maximum orders for non-seasonal and seasonal differencing were both set to 3. d) The stepwise method was not applied during the selection of the most appropriate model. e) The best-fitted ARIMA model was identified based on minimizing the Akaike information criterion with correction (AICc). f) Other parameters were left to be at default values. The Box–Ljung test was adopted to confirm whether the identified ARIMA model was valid. A key assumption of an ARIMA model fit is that residuals from the fitted model should be random. That is, residuals of the model are assumed to be white noise, and autocorrelation in them should be absent. The Box–Ljung test is commonly used to test for autocorrelation remaining in the residuals after fitting a model to a time series and is thereby suitable to test the above assumption of ARIMA model fit.

A segmented regression model was built using Stata software (version 16.0). Specifically, an itsa command specifying the newey option (note that this option is the default in the itsa command) was invoked. In the itsa command with the newey option, the parameters for the segmented regression model were estimated as per the ordinary least-squares principle, and Newey–West standard errors corresponding to the model parameters were specifically produced to handle possible autocorrelation and heteroskedasticity of the data. The actest command was also used to invoke the Cumby–Huizinga test to determine which lag order should be employed by the model to correctly account for the autocorrelation structure of the data (Linden, 2015). The Cumby–Huizinga test, which is used for testing autocorrelation in the errors of ordinary least-squares estimates for a single time series, helps uncover the appropriate lag order to be used because of its ability to determine whether serial correlation at the lag order specified exists (Baum and Schaffer, 2013). This is especially crucial for model fitting using the itsa command with the newey option, as the selection of lag order would significantly affect the accuracy of the model.

In all cases, the significance level (α) was set to 0.05, and *p* < 0.05 was considered statistically significant. A 95% confidence interval (CI) was produced and used as grounds for making CI-based statistical inferences.

## 3 Results

### 3.1 General description of the data collected

The data collected in this study are briefly summarized in Table 1.

**TABLE 1:** General description of the data collected.

Phase	Meropenem		Carbapenems		Penicillins/cephalosporins with beta-lactamase inhibitors		Carbapenem-replaced antimicrobials		Carbapenems plus carbapenem-replaced antimicrobials	
	Consumption^a^ (median, QR)	Expenditures^b^ (median, QR)	Consumption^a^ (median, QR)	Expenditures^b^ (median, QR)	Consumption^a^ (median, QR)	Expenditures^b^ (median, QR)	Consumption^a^ (median, QR)	Expenditures^b^ (median, QR)	Consumption^a^ (median, QR)	Expenditures^b^ (median, QR)
Pre-meropenem NCVBPP	6.1, 1.1	284.4, 71.6	12.0, 3.1	450.3, 146.0	105.2, 16.4	1044.4, 217.2	117.7, 20.4	1385.7, 214.3	127.1, 19.6	1836.0, 256.6
Post-meropenem NCVBPP	11.5, 2.6	116.7, 27.3	13.9, 2.7	206.1, 52.0	99.3, 11.9	1339.7, 542.4	103.9, 11.1	1431.8, 502.7	119.2, 17.2	1630.5, 607.3

Note:

^a^ expressed as DDD/1000 people/day.

^b^ expressed as thousand yuan; QR, quartile range.

### 3.2 Effects of the meropenem NCVBPP on the consumption of and expenditures for carbapenems

**FIGURE 1:**
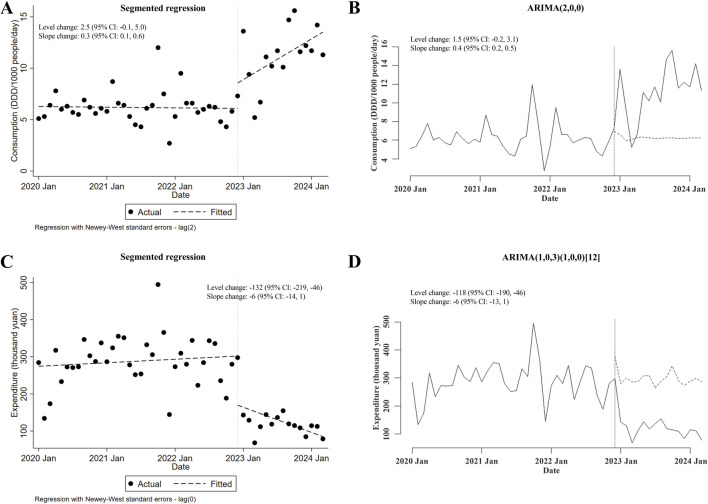
The meropenem NCVBPP increases the consumption of but reduces expenditures for meropenem. **(A, B)** Based on analysis using a segmented regression model **(A)** and an ARIMA model **(B)**, the consumption of meropenem was found to increase, following the meropenem NCVBPP. **(C, D)** Results of analysis using the segmented regression model **(C)** and the ARIMA model. **(D)** Indication of the effect of the meropenem NCVBPP on decreasing the expenditures for meropenem. Vertical dotted lines represent the date when the meropenem NCVBPP was initiated. Dashed lines in **(A, C)** indicate fitted values, while those in **(B, D)** indicate predicted values in the absence of the meropenem NCVBPP.

The influences of the meropenem NCVBPP on the consumption of and expenditures for both generic and branded meropenem used in inpatient departments were evaluated. As shown in Figures 1A, B, albeit the meropenem NCVBPP did not induce an immediate increase in the consumption of meropenem (level changes in the segmented regression and the ARIMA models were both statistically insignificant), it led to a significant change in the trend of meropenem consumption (slope changes in the segmented regression and the ARIMA models were 0.3 (95% CI: 0.1, 0.6) and 0.4 (95% CI: 0.2, 0.5) DDD/1000 people/day, respectively). Specifically, an evident trend that the consumption of meropenem rose with the passage of time was observed after but not before the meropenem NCVBPP, indicating a facilitating effect of the meropenem NCVBPP on the consumption of meropenem. In terms of the effects of the meropenem NCVBPP upon the expenditures for meropenem, it was found that the meropenem NCVBPP substantially decreased the expenditures for meropenem (level changes in the segmented regression and the ARIMA models were −132 (95% CI: −219, −46) and −118 (95% CI: −190, −46) thousand yuan, respectively; Figures 1C, D).

**FIGURE 2:**
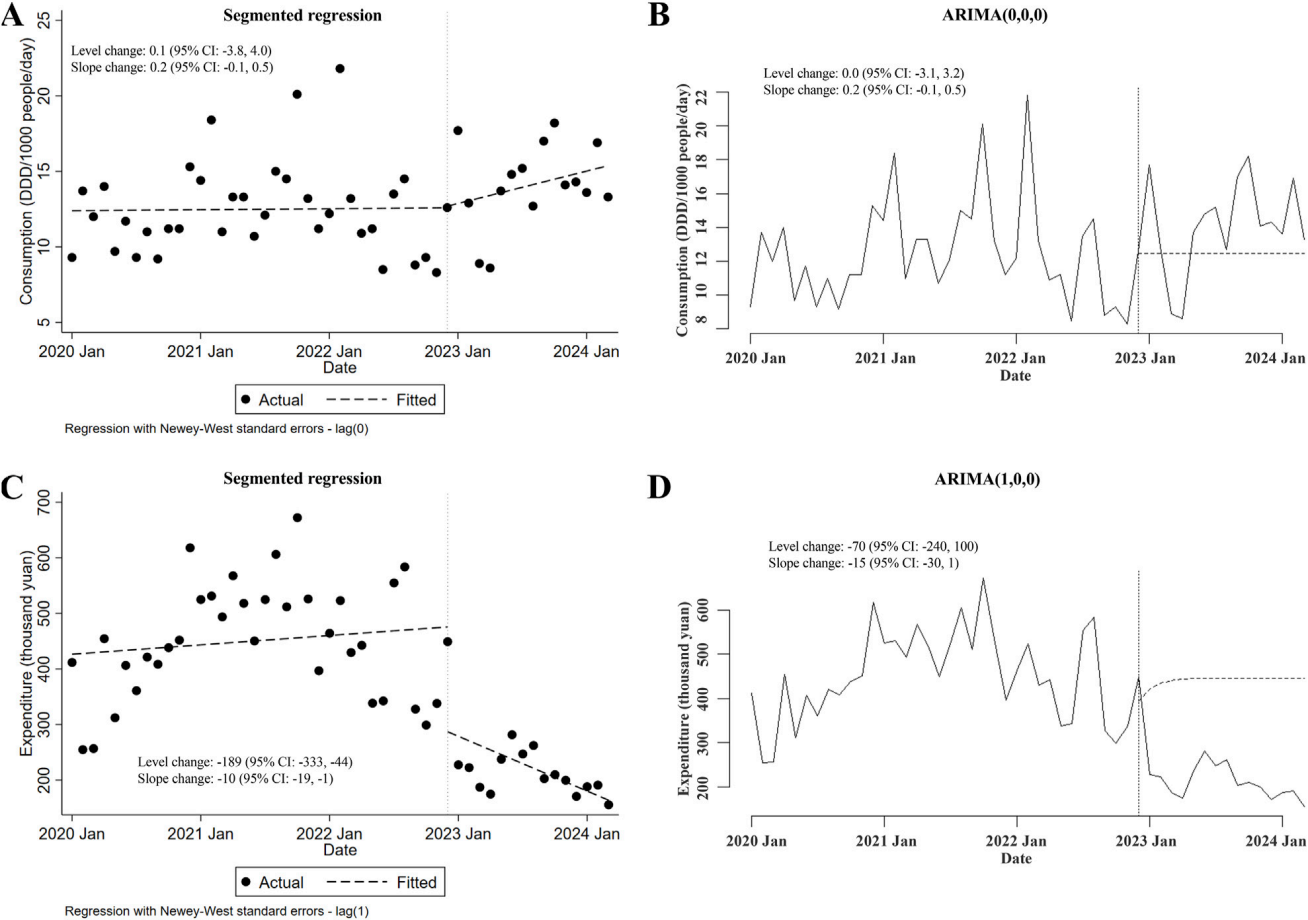
The meropenem NCVBPP has no effects on the consumption of carbapenems, and its effects on expenditures for carbapenems are unknown. **(A, B)** According to analysis using the segmented regression model **(A)** and the ARIMA model **(B)**, the meropenem NCVBPP failed to alter the consumption of carbapenems. **(C, D)** The impacts of the meropenem NCVBPP on the expenditures for carbapenems were unknown because the results of analysis using the segmented regression model **(C)** and the ARIMA model **(D)** contradicted each other. Vertical dotted lines represent the date when the meropenem NCVBPP was initiated. Dashed lines in **(A, C)** indicate fitted values, while those in **(B, D)** indicate predicted values in the absence of the meropenem NCVBPP.

Next, the possible changes in consumption of and expenditures for carbapenems prescribed for inpatients after the meropenem NCVBPP were also assessed. The NCVBPP of meropenem failed to affect the consumption of carbapenems (in either segmented regression or ARIMA, level and slope changes were both of statistical insignificance; Figures 2A, B). On the other hand, given that the results from ITSA using the segmented regression model contradicted those from ITSA using the ARIMA model (level and slope changes were both statistically significant in segmented regression, whereas neither of those were of statistical significance in ARIMA; Figures 2C, D), no conclusion could be made about the impacts of the meropenem NCVBPP on the expenditures for carbapenems.

### 3.3 Changes in consumption of and expenditures for carbapenem-replaced antimicrobials following the meropenem NCVBPP

**FIGURE 3:**
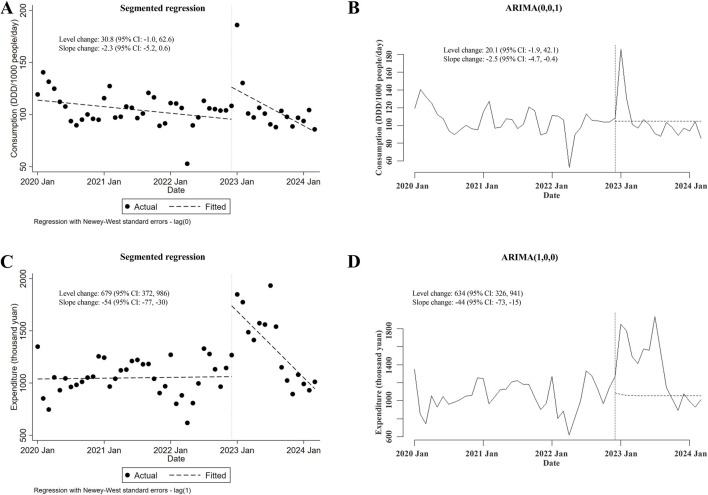
Alterations in the consumption of and expenditures for combinations of penicillins/cephalosporins with beta-lactamase inhibitors after the meropenem NCVBPP. **(A, B)** Due to the contradictory results from analysis using the segmented regression model **(A)** and the ARIMA model **(B)**, whether there was a change in the consumption of combinations of penicillins/cephalosporins with beta-lactamase inhibitors after the meropenem NCVBPP was not clear. **(C, D)** According to analysis using the segmented regression model **(C)** and the ARIMA model **(D)**, a sudden rise in the expenditures for combinations of penicillins/cephalosporins with beta-lactamase inhibitors occurred after the meropenem NCVBPP, followed by a reduction in the trend of expenditures for these antimicrobials. Vertical dotted lines represent the start date of the meropenem NCVBPP. Dashed lines in **(A, C)** indicate fitted values, while those in **(B, D)** indicate predicted values in the absence of the meropenem NCVBPP.

As concerned with the changes in consumption of the major type of carbapenem-replaced antimicrobials (that is, combinations of penicillins/cephalosporins with beta-lactamase inhibitors) prescribed for inpatients, no conclusion could be made because of the contradictory results from ITSA using the segmented regression model and those using the ARIMA model (slope changes in ARIMA but not in segmented regression were statistically significant; Figures 3A, B). As depicted in Figures 3C, D, with the implementation of the meropenem NCVBPP, a sudden rise in the expenditures for combinations of penicillins/cephalosporins with beta-lactamase inhibitors was observed (level changes in the segmented regression and the ARIMA models were 679 thousand yuan (95% CI: 372, 986) and 634 (95% CI: 326, 941) thousand yuan, respectively), followed by a decline in trend of these antimicrobial expenditures (slope changes in the segmented regression and the ARIMA models were −54 (95% CI: −77, −30) and −44 (95% CI: −73, −15) thousand yuan, respectively).

**FIGURE 4:**
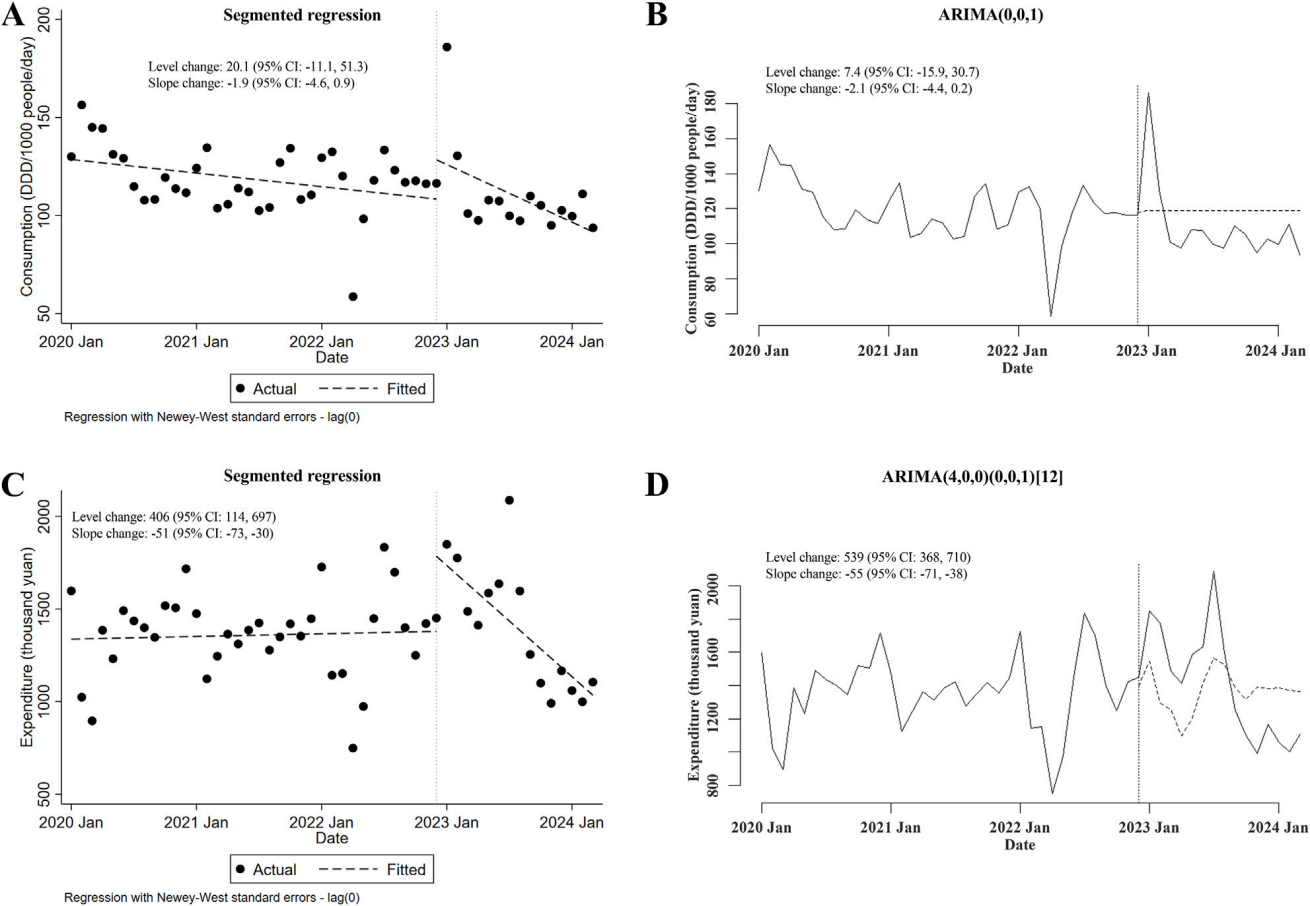
Changes in the consumption of and expenditures for all types of carbapenem-replaced antimicrobials following the meropenem NCVBPP. **(A, B)** Results from analysis using the segmented regression model **(A)** and the ARIMA model **(B)** indicated that the consumption of carbapenem-replaced antimicrobials did not change after the meropenem NCVBPP. **(C, D)** According to analysis using the segmented regression model **(C)** and the ARIMA model **(D)**, a transient increase in the expenditures for carbapenem-replaced antimicrobials appeared following the meropenem NCVBPP. Vertical dotted lines represent the date when the meropenem NCVBPP was initiated. Dashed lines in **(A, C)** indicate fitted values, while those in **(B, D)** indicate predicted values in the absence of a meropenem NCVBPP.

Regarding the alterations in the consumption of and expenditures for carbapenem-replaced antimicrobials (including both combinations of penicillins/cephalosporins with beta-lactamase inhibitors and cephamycins) utilized in the inpatient sector, it was shown that the consumption of carbapenem-replaced antimicrobials remained unchanged after the meropenem NCVBPP (in either segmented regression or ARIMA, level and slope changes were both of statistical insignificance; Figures 4A, B). Nevertheless, a transient increase in the expenditures for carbapenem-replaced antimicrobials following the meropenem NCVBPP was observed, which disappeared with time (level changes in the segmented regression and the ARIMA models were 406 thousand yuan (95% CI: 114, 697) and 539 (95% CI: 368, 710) thousand yuan, respectively; slope changes in the segmented regression and the ARIMA models were −51 (95% CI: −73, −30) and −55 (95% CI: −71, −38) thousand yuan, respectively; Figures 4C, D).

### 3.4 Changes in the consumption of and expenditures for carbapenems plus carbapenem-replaced antimicrobials after the meropenem NCVBPP

**FIGURE 5:**
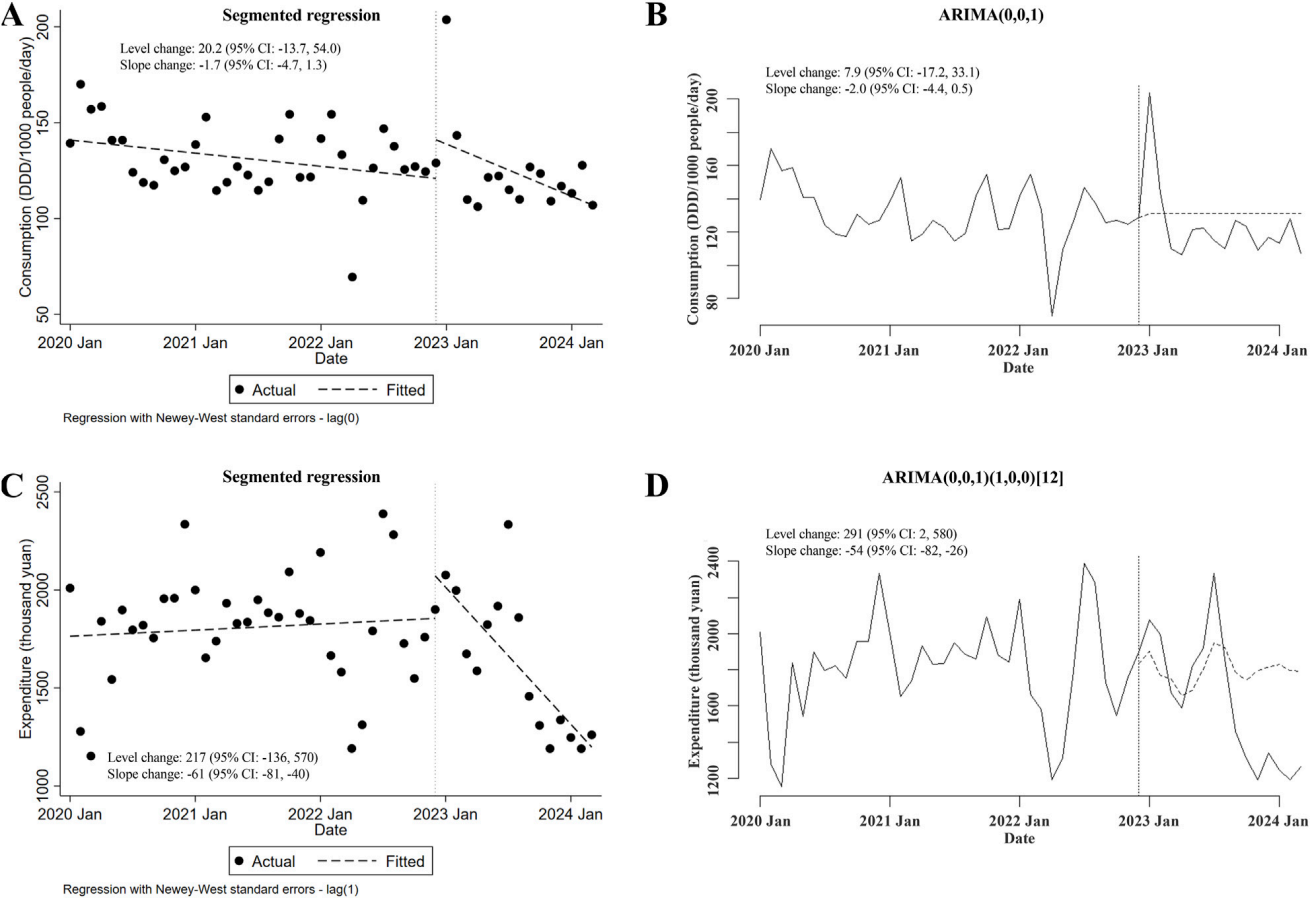
Changes in the consumption of and expenditures for carbapenems plus carbapenem-replaced antimicrobials after the meropenem NCVBPP. **(A, B)** Results from analysis using the segmented regression model **(A)** and the ARIMA model **(B)** showed that no alternation in the consumption of carbapenems plus carbapenem-replaced antimicrobials was observed after the meropenem NCVBPP. **(C, D)** According to analysis using the segmented regression model **(C)** and the ARIMA model **(D)**, expenditures for carbapenems plus carbapenem-replaced antimicrobials decreased following the meropenem NCVBPP. Vertical dotted lines represent the date when the meropenem NCVBPP was initiated. Dashed lines in **(A, C)** indicate fitted values, while those in **(B, D)** indicate predicted values in the absence of the meropenem NCVBPP.

Lastly, the potential changes in the consumption of and expenditures for carbapenems plus carbapenem-replaced antimicrobials used in inpatient departments after the meropenem NCVBPP were investigated. There was no change in total consumption of these antimicrobials following the meropenem NCVBPP (in either segmented regression or ARIMA, neither level nor slope changes were of statistical significance; Figures 5A, B). However, the overall expenditures for these antimicrobials substantially decreased after the meropenem NCVBPP, as suggested by an apparent downward trend in expenditures for these antimicrobials after the meropenem NCVBPP (slope changes in the segmented regression and the ARIMA models were −61 thousand yuan (95% CI: −81, −40) and −54 (95% CI: −82, −26) thousand yuan, respectively; Figures 5C, D).

## 4 Discussion

In the present study, the influences of the meropenem NCVBPP on the use of antimicrobials of interest were comprehensively studied. During the study design phase, the following items were taken into account to ensure the integrity of the design and the relative reliability of the results. First, in terms of the antimicrobials to be investigated, not only meropenem and carbapenem-type antimicrobials but also other antimicrobials, including combinations of penicillins/cephalosporins with beta-lactamase inhibitors and cephamycins, were considered as study subjects. Second, two measures reflecting the use status of specified antimicrobials (namely, consumption and expenditure) were accommodated. Third, both ITSA using the segmented regression model and ITSA using the ARIMA model were simultaneously employed to analyze the same set of collected data.

Diverse factors might influence antimicrobial use: a) changes in hospital policies. Changes in hospital policies may affect antimicrobial prescribing behavior. For example, the introduction of an antimicrobial stewardship program is associated with a change in antimicrobial prescribing behavior (Chavada et al., 2017). b) release of new treatment guidelines. New treatment guidelines may be effective in altering antimicrobial consumption (Schonherr et al., 2021). c) the COVID-19 pandemic. The COVID-19 pandemic has profoundly altered the landscape of healthcare, including antimicrobial use. The World Health Organization (WHO) has pointed out the extensive overuse of antibiotics during the COVID-19 pandemic (World Health Organization, 2024). d) changes in patient demographics. Changes in patient demographics (including age, comorbidities, socioeconomic status, etc.) could also play a significant role. For instance, an elderly population may have a higher susceptibility to infections and a larger exposure to antimicrobials than a younger cohort (Giarratano et al., 2018). e) the prevalence of specific infections. The prevalence of specific infections is a common contributor to changes in antimicrobial consumption. For example, during influenza season, the rate of antibiotic prescribing for patients with influenza has been shown to be high (Ciesla et al., 2004). f) hospital capacity. Hospital capacity is also a critical factor. In overcrowded hospitals with limited beds and resources, rapid patient turnover may be required, and physicians often prioritize speed over precision. The presence of these statuses is likely to result in premature cessation of antimicrobial courses or, conversely, overprescribing to cover all possible pathogens.

In spite of the efforts made to pursue the perfection of the study design, our study has several evident limitations. These limitations are listed as follows: a) All of the study results are based on the data of our hospital. Whether the results remain valid or not in healthcare settings beyond our hospital is unknown. In the future, large-scale studies based on local and even nationwide data involving the consumption of and expenditures for associated antimicrobials could be conducted to address this question. b) The potential effects of the abovementioned factors that might also influence antimicrobial consumption and expenditures were not examined or adjusted in our study due to the limitations of the ITSA methods used and the complexity or inability to determine the duration and/or consistency of actions imposed by these factors.

One notable result of this study is that the consumption of meropenem increased with the implementation of the meropenem NCVBPP. There are some underlying causes of the rising consumption of meropenem. Like other drugs subject to corresponding NCVBPPs, the bid-winning meropenem subject to the meropenem NCVBPP has passed through the bioequivalent assays, and its efficacy is thus thought to be consistent with the original counterpart. In addition, the bid-winning meropenem is much cheaper than the original counterpart and other carbapenem-type antimicrobials. These two features of the bid-winning meropenem make this drug more acceptable to doctors when they are making drug selections among the bid-winning meropenem, the original meropenem, and other carbapenem-type antimicrobials. On the other hand, healthcare security administration requires that the bid-winning meropenem subject to the meropenem NCVBPP be used preferentially in the hospitals. This is another factor contributing to the increased use of meropenem.

In sharp contrast with the effect the meropenem NCVBPP had on increasing the consumption of meropenem, its implementation resulted in a considerable decrease in the expenditures for meropenem, meaning that access to meropenem was further improved and a large savings on meropenem administration costs was achieved in patients subject to therapy with meropenem. In the context of the meropenem NCVBPP, although patients treated with meropenem would directly benefit from meropenem NCVBPP-induced lower administration costs, they may also be at risk of overexposure to meropenem, and the likelihood that they experience adverse reactions to meropenem would rise accordingly.

Our results that the meropenem NCVBPP could boost the consumption of meropenem itself reconciles with a published report that pointed out a promoting effect of an NCVBPP targeting cefuroxime on the use of cefuroxime (Yang et al., 2021). Such effect of an NCVBPP targeting antimicrobials could be referred to as a drawback of these NCVBPPs in view of the available evidence that there are causal associations between antimicrobial use and the emergence of antimicrobial resistance (Dellit et al., 2007; Llor and Bjerrum, 2014; World Health Organization, 2023). As with any other antimicrobials, meropenem’s use would incur the selection of resistant strains, thus contributing to the development of antimicrobial resistance. However, a direct correlation between the implementation of the meropenem NCVBPP and aggravated antimicrobial resistance was not evaluated in this study. Whether the meropenem NCVBPP has long-term effects on antimicrobial resistance is so far unknown; this is a question warranting further investigation. Antimicrobial resistance poses a serious threat to global public health (Prestinaci et al., 2015). An important measure to combat antimicrobial resistance is to prevent the overuse and misuse of antimicrobials.

Faced with the unwanted effects of the meropenem NCVBPP to increase the consumption of meropenem, stewardship targeting meropenem should be strengthened to ensure that this drug is rationally used in clinic. Fortunately, the effects of the meropenem NCVBPP to promote antimicrobial consumption were limited; only the use of meropenem itself was enhanced, and neither the consumption of carbapenems nor that of carbapenem-replaced antimicrobials altered following the meropenem NCVBPP. Based on these results, it is reasonable to speculate that the meropenem NCVBPP induced a dramatic change in the use proportion of various carbapenems. Specifically, with the implementation of the meropenem NCVBPP, the use of meropenem increased while that of carbapenems other than meropenem decreased. However, this speculation found no favor with our analysis on the consumption data of carbapenems other than meropenem because the related ITSA using a segmented regression model and the ITSA using the ARIMA model obtained conflicting results (the result from ITSA using a segmented regression model indicates a decrease in the consumption of carbapenems other than meropenem following the meropenem NCVBPP while that of ITSA using the ARIMA model does not. Data not shown).

Our result that the use of carbapenem-replaced antimicrobials remained unaffected following the meropenem NCVBPP suggests that there is no transition of use preference from carbapenem-replaced antimicrobials to carbapenems after the meropenem NCVBPP. This is a welcome outcome, which, to some extent, means that our antimicrobial stewardship toward carbapenem-replaced antimicrobials and carbapenems is effective. In China, antimicrobials used in clinic are divided into three categories: unrestricted use, restricted use, and special use (Ministry of Health of the PRC, 2012). In Fujian Province, where our hospital is located, almost all types of carbapenems are categorized in the special use category of antimicrobials, while most carbapenem-replaced antimicrobials belong to the restricted use category. Compared to carbapenem-replaced antimicrobials, carbapenems are required to be used more prudently and are viewed as key targets of antimicrobial stewardship programs in medical institutes to preserve their effectiveness.

The meropenem NCVBPP could bring a massive drop in the expenditures for meropenem. This is not surprising because the procurement subject of the meropenem NCVBPP, generic meropenem, is much cheaper in sales price than the replaced original counterpart. A decline in the expenditures for carbapenems plus carbapenem-replaced antimicrobials was also seen after the meropenem NCVBPP, yet the occurrence of this phenomenon was not only attributed to the implementation of the meropenem NCVBPP because other NCVBPPs targeting combinations of penicillins with beta-lactamase inhibitors exist. In August 2023, an NCVBPP targeting piperacillin/tazobactam and amoxicillin/clavulanate was implemented. Undoubtedly, the implementation of these NCVBPPs could lead to a decrease in the expenditures for the corresponding antimicrobials. It presents as a potential confounding factor whose confounding effects could not be adjusted by the ITSA. For the same reason, another obtained result showing a transient increase in the expenditures for carbapenem-replaced antimicrobials after the meropenem NCVBPP does not necessarily mean that the meropenem NCVBPP does affect the expenditures for carbapenem-replaced antimicrobials.

In sum, we show in this study that, as far as our hospital is concerned, implementing the meropenem NCVBPP increased consumption but reduced expenditures for meropenem, whereas it has no effects on the overall consumption of carbapenems.

## Data Availability

The raw data supporting the conclusions of this article will be made available by the authors, without undue reservation.

## Abbreviations

ARIMA, autoregressive integrated moving average; ITSA, interrupted time series analysis; NCVBPP, national centralized volume-based procurement policy.
